# Practical Chemistry for Students and Physicians—Chapter Third

**Published:** 1883-09

**Authors:** 


### PRACTICAL CHEMISTRY FOR STUDENTS AND PHYSICIANS—CHAPTER THIRD.

(Continued from August Register, Page 696.)

It was stated in the last chapter that the way is now open for the consideration of such characteristics of the elements and their compounds, as are interesting and important.

*Physical Condition of the Elements.*—Of the thirty-nine elements of medical and pharmaceutical interest, thirty-two are solid, two liquid and five gaseous—fluorine, the least useful among them, being regarded as a gas. So that the three natural conditions, in one or the other of which all matter is found, have each its representatives among the elementary bodies—solidity, liquidity and gaseity.

*Metals and Non-Metals.*—A more general and therefore more useful classification of these bodies, is their division into metals and non-metals. In the catalogue of elements to which we are restricted, there are twenty-seven metals and twelve non-metals. Of the twenty-seven metals, one is a liquid—mercury; the remainder are all solids.

Among the non-metals appear five gases, six solids and one liquid, bromine. Of the two liquid elements, one is a metal and the other a non-metal.

*The Metalic and Non-Metalic Solids.*—The names of the elementary solids, whether metals or non-metals, are carbon, iodine, sulphur, phosphorus, potassium, sodium, ammonium, barium, calsium, magnesium, iron, aluminnium, zinc, arsenicum, antimony, copper, lead, silver, boron, silicon, lithium, strontium, cerium, cromium, manganese, cobalt, nickel, tin, gold, platinum, bismuth and cadmium —thirty-two.

*The Gases all Non-Metals.*—The elementary gases are oxygen, hydrogen, nitrogen, chlorine, and probably fluorine.

*Study of the Gases.*—In order to a satisfactory investigation of these bodies, it is necessary to generate and collect them. This introduces the consideration of apparatus and certain methods of chemical manipulation.

*Apparatus for Generating and Storing Oxygen and Other Gases.*—These are quite simple, and may be inexpensive. Three or four test-tubes, or wide-mouthed bottles, a piece of glass tubing, a perforated cork, a spirit or Bunsen's lamp, and a common basin of water. Technically the basin is the pneumatic trough of physical chemistry.

*Preparation of Oxygen and Use of the Apparatas.*—The usual source of oxygen, either for experimental or practical purposes, is potassium chlorate, $KClO_3$, heated with one fourth its weight of manganese dioxide, $Mn\ O_2$, sometimes called black oxide of manganese.

Weigh out fifteen grains or one gramm of the $KClO_3$ and about one-fourth as much of the $Mn\ O_2$. Mix and place the mass in a test-tube, well stopped with a perforated cork, to which a glass tube has been adjusted, and so bent as to conveniently deliver the gas as fast as it is generated into a test-tube or bottle prepared to receive it over the basin of water.

The receiving bottle or tube must, of course, be first filled with water, and then being inverted, held with its mouth just beneath the water so as to exclude the air. Heat is now applied to the mixture in the generating tube, and the gas quickly begins to pass from this tube into the receiver, displacing the water from it as it ascends, till the receiver is full of the gas. This is known when there is no more water in the receiver, or when the gas begins to bubble up in the basin outside of the receiving tube.

The apparatus employed in the above process is shown in fig. 1.

*History of Oxygen.*—The discovery of this gas by Dr. Priestley in 1774, was the most important scientific event of the eighteenth century. Priestly named it dephlogisticated air; for he did not then know, and died without knowing, the real nature and scientific value of the substance he had discovered. Seven years after the discovery of oxygen, Lavoisier, a great French chemist, overthrew the old theory of phlogiston, and comprehending better the character of the new gaseous element, he named it oxygen, from the Greek oxus and gennao, acid-former. It will be seen, however, that Lavoisier was himself greatly in error in supposing oxygen to be the great acid former of nature. The name, as expressive of this fact, belongs of right, and is now in effect, universally assigned to another gaseous element—hydrogen.

*Occurrance and Diffusion of Hydrogen.*—Oxygen is the most abundant of the elements. In the form chiefly of oxides it constitutes two-thirds of the earth's bulk. In a free state it forms one-fifth of the ocean of atmosphere. Eight-ninths of all water is hydrogen. Of all minerals, one-half is oxygen ; three-fourths of the weight of animals and four-fifths of the weight of all vegetables is oxygen.

*Chemical Properties of Oxygen.*—The chemical activity of oxygen is intense. Urged by various degrees of heat, it enters into chemical combination with every known element except flourine. The discovery of oxygen explained the phenomenon of ordinary combustion—the chemical combination of oxygen with inflammable substances, attended with light and heat. The principal of those inflammable substances are, hydrogen, sulphur, charcoal or carbon, phosphorus and sodium. The compounds resulting are, of course, oxides· Pure oxygen is also respirable. Its quantivalence is 2.

*Physical Properties of Oxygea.*—This gae has neither taste nor smell, and is so transparent as to be invisible. Air being 1, its weight is 1.105. Hydrogen being 1, its weight is 16. One liter of it weighs 1.43 grains. Of all the elementary bodies, oxygen is the most negitive in its electro-chemical relations.

*Catalysis in the Preparation of Oxygen.*—It was directed to mix manganese dioxide with the potassium chlorate in the preparation of oxygen. Why? This question has never been satisfactorily answered. The part played in the process by the manganese, whatever it may be, is called in chemistry "catalysis;" that is, contact action from the Greek *kataleusis* dissalution.

*Preparation of Hydrogen.*—It has been seen that water is rich in hydrogen; two-thirds of every volume of water is hydrogen; water, therefore, is the principal source of hydrogen gas. There are several methods by which it may be prepared from water, but the best is that by the action of the metal zinc upon cold, dilute sulphuric acid The same apparatus, used in preparing oxygen, may be employed here, omitting the lamp, as no heat is required. Drop about a gram of zinc clippings into a test tube, better into a wide-mouthed quinine bottle and pour in just enough water to cover them. Now add about one fifth as much of strong sulphuric acid as there is water and adjust the stopper with the delivery tube. The gas is usually evolved at once, and may be collected, like the oxygen, in tubes or receivers over the basin of water. The first bottle full of the gas should be rejected, being mixed with the air previously in the apparatus, and therefore not pure hydrogen. Besides, if it should be ignited in that mixed state an explosion would follow.

*Chemical Properties of Hydrogen.*—If hydrogen be heated to 932° F. it will instantly combine with oxygen, involving much heat and some light; it is therefore combustible. Burning hydrogen evolves more heat, per gram, than any other known substance. This heat will raise 1°, 34,462 grams of water, and in mechanical energy is equal to the work of raising 14,605,826 grams one meter high. Hydrogen is not a supporter of combustion and of respiration; no candle can burn in it, and no animal breathe it and live. Hydrogen is the standard of quantivalence and of atomic weight.

*Physical Properties of Hydrogen.*—Like oxygen, hydrogen has neither taste nor smell, and is transparent. Its spec. gr. is 0.0693, being 14.45 times lighter than air; it is the lightest substance known. It is 11,000 times lighter than water. Hydrogen is the standard of density—of molecular weight and volume. A liter of it weighs 0 0896 of a gram.

